# O10.7. INVESTIGATING THE MECHANISMS UNDERLYING THE BENEFICIAL EFFECTS OF ESTROGENS IN SCHIZOPHRENIA

**DOI:** 10.1093/schbul/sby015.259

**Published:** 2018-04-01

**Authors:** P J Michael Deans, Carol Shum, Leo Perfect, Marco Conforti, Rodrigo Duarte, Sagnik Bhattacharyya, Nicholas Brandon, Jack Price, Deepak Srivastava

**Affiliations:** 1 Institute of Psychiatry, King’s College London; 2 AstraZeneca Neuroscience iMED

## Abstract

**Background:**

Estrogens, in particular 17β-estradiol (estradiol) have repeatedly been shown to exert powerful influences over cognitive function, and in particular, on a range of cognitive behaviours associated with neurodevelopmental disorders. This includes depressive and anxious behaviours as well as learning and memory (including working memory). These cognitive enhancing effects have been shown to be dependent on increases in the number of dendritic spines as well as alterations in glutamate receptor transmission and regulation of synaptic protein expression. Modulation of these synaptic functions can result in long-term increases in synaptic connectivity.

Interestingly, there is growing evidence that estrogenic-based compounds may have a positive effect in the treatment of a number of neuropsychiatric disorders, including schizophrenia. Importantly, recent clinical studies have demonstrated that adjunct treatment with estradiol or the selective estrogen receptor modulator (SERM) raloxifene, ameliorates positive and negative symptoms and improves working memory and attention deficits in male and female schizophrenic patients. However, it has been argued that estrogenic-based compounds are not an effective treatment option owing to potential serve side effects associated with long-term administration. It is, however, of note that the precise mechanisms that underlie the positive effects of estradiol, or estrogenic-based compounds, in this disease are currently unclear. Therefore, determining how estradiol exerts its positive effects in health as well as in disease, will aid in the development of safer and more effective estrogenic-based compounds.

**Methods:**

Here, we have used human induced pluripotent stem cell (iPSC)-derived from healthy or patients diagnosed schizophrenic but with no common genetic background to study the potential mechanism that may underlie estrogens beneficial effects in disease. iPSCs were differentiated into young, developing, cortical neurons using well established methods. First, we assessed the ability of estrogens to modulate key neuronal and synaptic structures as well as synaptic and inflammatory genes. Next, we assessed the expression and distribution of synaptic proteins were determined in both healthy iPSC-neurons and patient iPSC-neurons (from 3–6 individuals from each group). Subsequently, using a pharmacological approach, we have explored the ability of estrogens to rescue cellular and molecular deficits in iPSC-neurons derived from schizophrenic patients.

**Results:**

Both healthy and patient iPSC differentiated into neuroepithelium, neural progenitors cells and finally into TBR1- and EMX1-positive neurons efficiently. Assessment of synaptic protein expression revealed reduced expression of key synaptic proteins involved in excitatory transmission compared to control lines. When healthy iPSC-neurons were treated with a range of estrogenic compounds, we observed a robust increase in the expression of key synaptic protein including GRIN1 and DGL4. Consistent with previous reports, patient iPSC-neurons displayed reduced synaptic protein expression compared with healthy iPSC-neurons. Critically, when patient iPSC-neurons were treated with 17β-estradiol or raloxifene, we observed an increase in synaptic protein expression to a level similar to that observed in untreated healthy iPSC-neurons.

**Discussion:**

These data are the first to demonstrate that estrogens are capable of regulating synaptic proteins in human neurons taken from patients diagnosed with schizophrenia. Collectively, we hope these data will help us understand how estrogens may confer their positive effects in psychiatric disorders.

